# Heart and Stroke Foundation of Ontario (HSFO) high blood pressure strategy's hypertension management initiative study protocol

**DOI:** 10.1186/1472-6963-8-251

**Published:** 2008-12-10

**Authors:** Sheldon W Tobe, Margaret Moy Lum-Kwong, Nancy Perkins, Shirley Von Sychowski, Rolf J Sebaldt, Alex Kiss

**Affiliations:** 1 Division of Nephrology, Suite A240, Sunnybrook Health Sciences Centre, 2075 Bayview Avenue, Toronto, Ontario, Canada; 2 Heart and Stroke Foundation of Ontario, 2300 Yonge Street, Suite 1300, Toronto, Ontario, Canada; 3 Centre for Evaluation of Medicines, 105 Main Street East, Hamilton, Ontario, Canada; 4 Institute for Clinical Evaluative Sciences, 2075 Bayview Avenue, Toronto, Ontario, Canada

## Abstract

**Background:**

Achieving control of hypertension prevents target organ damage at both the micro and macrovascular level and is a highly cost effective means of lowering the risk for heart attack and stroke particularly in people with diabetes. Clinical trials demonstrate that blood pressure control can be achieved in a large proportion of people. Translating this knowledge into widespread practice is the focus of the Hypertension Management Initiative, which began in 2004 with the goal of improving the management of this chronic health condition by primary care providers and patients in the community.

**Methods:**

This study will test the effect of a systems change on the management of high blood pressure in real world practice in primary care in Ontario, Canada. The systems change intervention involves an interprofessional educational program bringing together physicians, nurses and pharmacists with tools for both providers and patients to facilitate blood pressure management. Each of two waves of subjects were enrolled over a 6 month period with the initial enrollment between waves separated by 9 months. Blood pressure will be measured with the BpTru ^® ^automated blood pressure device. To determine the effectiveness of the intervention, a before and after analysis within all subjects will compare blood pressure at baseline to annual measurements for the three year study. To assess whether the intervention has an impact on blood pressure control independent of community trends, a betwen group comparison of baseline blood pressures in the delayed wave will be made with the immediate wave during the same time period, so that the immediate wave has experienced the intervention for at least 9 months. The total enrollment goal is 5,000 subjects. The practice locations include 10 Family Health Teams (FHTs) and 1 Community Health Centre (CHC) and approximately 49 primary care physicians, 15 nurse practitioners, 37 registered nurses and over 150 community pharmacists across the 11 communities throughout the province of Ontario. The 11 primary care sites will be divided into immediate and delayed groups based on geography and the use of an electronic versus a traditional chart patient record.

**Discussion:**

Initial consideration was given to randomizing the groups, however, for a number of reasons, this was deemed to not be possible. In order to ensure that the sites in the immediate intervention and delayed intervention groups are not different from each other, the sites will be assigned to the intervention groups manually to ensure a distribution of the variables as evenly as possible.

Given that HSFO approached this particular group of health care providers to participate in a program relating to hypertension, this may have heightened their awareness of the issue and affected their management of patients with hypertension. Thus, data will be collected to allow an assessment of previous practice patterns and determine any impact of the Hawthorne Effect.

**Trial registration:**

Clinicaltrials.gov NCT00425828

## Background

### Hypertension: Its Burden of Disease

Hypertension is a major risk factor for cardiovascular morbidity and mortality, which in Canada, is the diagnostic category ranked as the highest for drug expenditures[1]. The economic burden of chronic disease in Ontario accounts for 55% of direct and indirect total health costs in Canada [1]. With the current demographic changes in the province of Ontario due to the aging of the 'Baby Boomer' generation, an unusually large cohort born between 1946 and 1965, provincial health plans are grappling with how to plan for the 'silver tsunami' as this generation become senior citizens over the next 23 years. Hypertension, also referred to as the 'silent killer', is a significant chronic health condition, and is prevalent in over 1 out of 5 adult Canadians [2]. Despite being the number one diagnosis listed for patient visits, a substantial proportion of people remain unaware of their hypertension or have not achieved treatment targets [3]. Data compiled by the Heart and Stroke Foundation of Ontario indicate that consumers do not take hypertension seriously, that physicians are not consistently applying clinical practice guidelines related to diagnosis and treatment targets, that patients are not adhering to prevention and treatment recommendations and that physicians lack time and skill to counsel patients about needed lifestyle modifications. Clinical trials demonstrate the efficacy and safety of blood pressure control and clinical practice recommendations provide guidance to achieve blood pressure targets [4]. However, to achieve the benefits from blood pressure control across the entire population, without expending an ever larger fraction of primary care physician's time to the management of this chronic disease, a new paradigm is required. The goal of the Heart and Stroke Foundation of Ontario's Hypertension Management Initiative is a sustainable chronic disease management model for blood pressure control, to economically realize the public health benefits of achieving blood pressure targets and the attendant reduction of heart attack and stroke.

### The High Blood Pressure AIM at HSFO

In 2004, the Heart and Stroke Foundation of Ontario (HSFO) Board of Directors approved a new strategy for making an impact in the fight against heart disease and stroke. This strategy, which is in addition to its ongoing commitment to research and health promotion, would begin to invest in 'Areas of Investment in Mission' (AIMs) as another way to achieve its mission impact.

**Figure 1:**
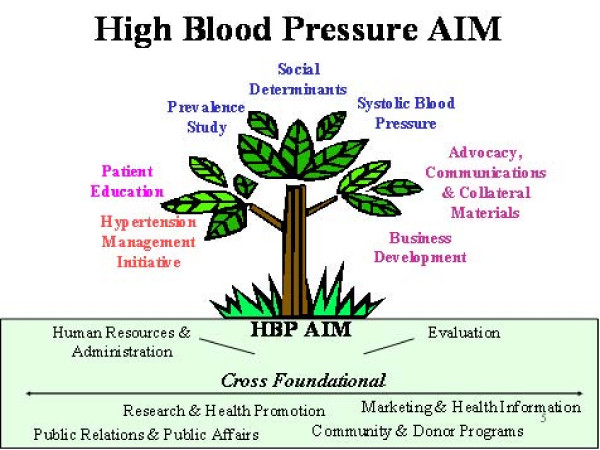
**High Blood Pressure Strategy**.

One of the major components of the HBP AIM Strategy is the Hypertension Management Initiative (HMI) (see Figure 1). The Hypertension Management Initiative focuses on what is acknowledged to be a critical issue in high blood pressure – improving the management of this chronic health condition by primary care providers (doctors, nurse practitioners, nurses and community pharmacists) and patients. Working with several key partners, including the Ontario College of Family Physicians, the Registered Nurses Association of Ontario, and the Ontario Pharmacists' Association, the HMI implements and evaluates a local systems change educational approach designed to enhance physician, nursing and pharmacist approaches to high blood pressure detection, intervention, and follow up measures. In addition, it is intended to improve communication and collaboration between these healthcare providers thus positively impacting patient self management.

The pilot (alpha) phase, "Primary Care Partnerships for Blood Pressure Reduction", was funded by the Ministry of Health and Long-Term Care, in the Province of Ontario, through the Primary Healthcare Transition Fund (PHCTF) and was completed in July 2006. The alpha phase evaluation revealed a statistically significant increase in screening (BP was measured at 45.7% of visits vs. 27.8% pre-program) and diagnosis of hypertension (11% vs. 4.8% pre-program) as well as a trend towards improved follow up and better control (31% of non-diabetic patients controlled vs. 25% pre-program, 58% of diabetic patients controlled vs. 46% pre-program). However the sample size was small at approximately 296 patients. The second phase, termed, the 'HMI Beta Phase', is fully funded by HSFO. The trial will be managed by the Heart and Stroke Foundation of Ontario using a team of experts. To date, eleven provider sites have been recruited across the province.

### Key Objectives of HMI – Beta Phase

The HMI Beta Phase is designed to improve patient outcomes with respect to the control of blood pressure. There are two key elements to the initiative. Firstly, it is designed to improve provider practices and management of essential hypertension. Secondly, it is designed to improve provider-patient interactions and patient self-management of hypertension.

The HMI Beta Phase will achieve these objectives by incorporating various strategies, including inter-professional education, practice outreach and facilitation, an inter-professional healthcare provider toolkit and a patient toolkit (see Table 1). Together, these tools and interventions are intended to enhance collaboration amongst primary healthcare professionals (physicians, nurses, nurse practitioners and community pharmacists) and between healthcare professionals and patients to enhance patient self management of blood pressure and improve the management and control of hypertension. The HMI beta phase also aims to determine and measure the effectiveness of the tools and improve best practice use of the current Canadian Hypertension Education Program (CHEP) recommendations [5] with a larger sample size of healthcare providers and patients than in the HMI Alpha phase. The HMI beta phase aims to measure the effectiveness of this initiative on a larger sample size by roughly quintupling the number of healthcare providers and patients.

**Table 1:** Aspects of the HMI beta phase

• interactive, inter-professional education workshops, reflective exercises
• practice outreach and support to healthcare providers to support and assist in the integration of the tools and interventions in their practices
• inter-professional, evidence-informed healthcare provider toolkit that incorporates clinical reminders and prompts and practice audit feedback and aids inter-professional communication
• evidence-informed, leading edge patient toolkit that provides information and education and supports patient self-management.

The HMI Beta phase is a three-year initiative, which will end in 2010 to determine the effectiveness of the toolkits and interventions on the management and control of hypertension.

## Methods and design

### Overview of the Evaluation Methodology

#### Evaluation Design

To evaluate this initiative, a prospective delayed intervention design has been selected. See Table 2 for key evaluation indicators. Although all sites ultimately will receive the intervention, six of the sites will be assigned to receive the intervention immediately (these sites will be referred to as "immediate intervention group"), while the remaining five sites will receive the intervention after several months, but will serve as a comparison during this delay period (these sites will be referred to as "delayed intervention group"). Further details regarding assignment to the immediate and delayed intervention groups will be discussed in a later section.

**Table 2:** Key Evaluation Indicators of Interest

**Sustainability of the Hypertension Management Initiative program**
◦ Development and usage of information management systems to improve and standardize high blood pressure patient care and treatment and to improve outcomes
◦ Statement of intention by participating healthcare providers to continue using key tools and learnings from the Hypertension Management Initiative.

**Improved hypertension management practices by healthcare providers participating in the Hypertension Management Initiative**
◦ Improved knowledge regarding best practices in management of patients with high blood pressure/hypertension
◦ Improved consistency with current CHEP recommendations in time elapsed from first high blood pressure reading to hypertension diagnosis
◦ Improved healthcare provider medication prescribing for hypertension patients consistent with current CHEP recommendations
◦ Improved assessment of medication adherence and side effects in high blood pressure/hypertension patient visits
◦ Improved assessment of global cardiovascular disease risk in patients with high blood pressure/hypertension
◦ Improved assessment of high blood pressure/hypertension patients' lifestyle risk factors and readiness to undertake lifestyle modifications
◦ Improved provision of support and advice to motivate high blood pressure/hypertension patients to make lifestyle recommendations
◦ Improved follow-up of high blood pressure/hypertension patients consistent with current CHEP recommendations
◦ Improved quantity and quality of inter-professional collaboration in management of patients with high blood pressure/hypertension.

**Improved control of blood pressure among patients reached**
◦ Reduced blood pressure readings of hypertension patients
◦ Increased proportion of patients with hypertension with blood pressure controlled to target
◦ Improved adherence of hypertension patients to prescribed hypertension medication(s)
◦ Improved adherence of high blood pressure/hypertension patients to their selected lifestyle changes

#### Rationale for the Evaluation Design

The reasoning for this delayed approach is that it helps to minimize the potential for threats to internal validity, which are defined as alternate explanations for outcome results other than the impact of the intervention itself. The inclusion of the delayed group controls for outside influences that may occur over time such as educational materials, new research results relating to hypertension or the release of new clinical practice recommendations.

As shown in the timeline (Table 3), the immediate intervention sites will receive the intervention over a 9 month period between Time 2 and 3, including the workshop and all tools developed, whereas the delayed intervention sites will not begin receiving the intervention until Time 3. Thus, between Time 2 and Time 3, it is hypothesized that healthcare providers in the immediate intervention sites will begin to use the tools developed by HSFO and to optimize their management of hypertensive patients. Healthcare providers in the delayed intervention sites will not have received the intervention and are expected to continue with treatment as usual. It is planned that blood pressure measurements will be conducted at Time 3 for patients in both the immediate intervention (after 9 months of the intervention) and delayed intervention (at their baseline) sites. Since the patients in the immediate intervention sites will have been enrolled nine months earlier, it is expected that their blood pressures in follow up visits will be better controlled (due to improvements in provider hypertension management practices stemming from their participation in the initiative and usage of tools developed) than those of their counterparts in the delayed intervention sites who will be providing their baseline BP reading at Time 3 when they are prepared to start the intervention.

**Table 3:** HMI Beta Phase Evaluation Timeline

	**Time 1**	**Time 2**	**Time 3**	**Time 4**	**Time 5**
**Immediate Intervention**	*Measurement:* • BP readings (sphygmom.) • site characteristics • practice patterns re. HTN management	*Receive intervention*: • workshop • tools enrolment of patients *Measurement*: • baseline BP readings (BpTRU)	(continue to receive site support for intervention) *Measurement*: • follow up visit • BP readings (BpTRU)	(continue to receive site support for intervention) *Measurement:* • follow up visit • BP readings (BpTRU)	*Measurement:* • follow up visit • BP readings (BpTRU) • practice patterns re. HTN management

**Delayed Intervention**	*Measurement*: • BP readings (sphygmom.) • site characteristics • practice patterns re. HTN management	treatment as usual	*receive intervention:* • workshop • tools enrolment of patients *measurement*: • baseline BP readings (BpTRU)	(continue to receive site support for intervention) *Measurement:* • follow up visit • BP readings (BpTRU)	*Measurement:* • follow up visit • BP readings (BpTRU) • practice patterns re. HTN management

### HMI Beta Phase Action Pathway

#### In-Depth Initiative Components

##### Inter-professional Collaboration: Coach Selection and Training

In order to facilitate inter-professional collaboration, which is an integral part of this initiative; coaches from each of the three professions (Physicians, Nurses/Nurse Practitioners, and Pharmacists) at each of the sites were identified to function as enablers and to provide a link between the enrolled sites and the HSFO. The coaches were identified at each of the sites and invited to attend an orientation session for this role. During the course of the HMI, regular communication with the participating healthcare providers will be maintained including periodic meetings of practitioners will continue for the three years of the project.

##### Site Selection and Provider Recruitment

A total of 11 primary care practice sites have been recruited for the HMI Beta Phase. At these sites, there are a total of 49 family physicians and 15 nurse practitioners, each of whom is asked to recruit 100 to 200 patients per full time equivalent rostering physician/nurse practitioner, a potential for up to 8,000 patients. Criteria for site recruitment are found in Table 4.

**Table 4:** Criteria for site recruitment

• Organized primary healthcare team configuration (e.g. Family Health Teams, Family Health Networks, Community Health Centers) that have a capitated or salaried fee structure. (for definition of these types primary care practice groups, see footnote)
• Geographic vicinity to reflect Ontario's diverse needs (northern, urban, and rural).
• Minimize cross research bias. Each primary healthcare team selected will not be participating in any other hypertension studies

Community pharmacists were recruited based on a convenience sample of the community pharmacies neighboring each of the participating medical clinics. To date there have been more than 150 pharmacists recruited. The final breakdown of potential participating healthcare providers by site is provided in Table 5. Each healthcare provider will have access to a variety of tools and resources to assist in improving management of hypertensive patients. There are no risks anticipated from a healthcare providers' participation in this study.

**Table 5:** Recruited HMI Beta Phase Sites

**Pilot Site Community** **(listed by number)**	**Type of** **Primary Care Site**	**Medical** **Record Type**	**# Physicians** **+ NP**	**# NP**	**# Nurses** **(RN and RPN)**	**# Pharmacists**
1	FHT	Paper	16	1	0	21

2	FHT	EMR	1	2	2	6

3	FHT	EMR	5	1	3	25

4	FHT	EMR	10	0		1

5	CHC	Paper	1	4	3	12

6	FHT	EMR	3	1	2	13

7	FHT	EMR	9	1	2	20

8	FHT	Paper	2	1	1	4

9	FHT	Paper	1	2	1	16

10	FHT	Paper	10	1	6	7

11	*FHT*	EMR	5	2	4	7

Total			63	17	25	132

FHT = Family Health Team, CHC = Community Health Centers

A series of tools were developed and piloted in the in the alpha phase of this initiative. These include specific tools for physicians, nurse practitioners and pharmacists as well as tools for patients. Descriptions of these tools are given in Table 6.

**Table 6:** HMI Healthcare Provider and Patient Toolkits

**Toolkit**	**Tool Name**	**Description**	**Target Audience**
**Provider Toolkit**	1. Rx Dx Pads (with Diagnosis check off)	Communication tool for physicians to provide pharmacists diagnosis information with prescription allowing pharmacist to counsel patient appropriately and reinforce physician's messages regarding treatment adherence. It will be available in both paper and electronic form according to site preference.	Physician/Nurse Practitioner

	2. Proper Methods to Take Blood Pressure	A summary of directions for providers and patients on how to take blood pressure; includes step by step instructions for patients before and during BP measurements to ensure a correct BP reading.	

	3. Bp TRu 300	Automated office blood pressure measurement devices.	

	4. Blood Pressure Pocket Guide	An easy to use reference, pocket guide with Blood Pressure Risk chart/BMI calculator, waist circumference risk and stages of Change and counselling information to help your patients with their identified lifestyle modification. Patients also like the visual risk mapping format of the charts.	Provider

	5. HBP Flow Sheet & Assessment Form	Charting tool to record patient's progress with HBP care plan over time. This tool will remain in the patient's chart and will be used in paper version only in paper based sites. That form will be also faxed to an optical recognition site that will digitize the data and send it to a secure web-based custom data set. EMR (electronic medical record) sites will be sending the data directly to the same secure web based custom data set through a secure link.	Provider

	6. Confidential Provider Practice Audit Reports	Regular confidential practice reports are generated from the web-based data set and provided to the healthcare providers, namely physicians and nurse practitioners. The reports provide confidential, anonymous comparisons of one's practice to other participating healthcare providers and sites. The dataset is hosted on a PHIPA compliant server.	Physician/Nurse Practitioner

	7. RNAO-HSFO Nursing Best Practice Guideline, Nursing Management of Hypertension	A nursing best practice guideline collaboratively developed by the Registered Nurses' Association of Ontario and the Heart and Stroke Foundation of Ontario, with funding support from the Ontario Ministry of Health and Long-Term Care. This guideline has been endorsed by CHEP.	Nurses, Nurse Practitioners

	8. CHEP Recommendations	Canadian Recommendations for the Management of Hypertension, published yearly by the Canadian Hypertension Education Program(CHEP)	

	9. 'Reach Your Goal in Your Blood Pressure Control' Fact Sheet	The Reach Your Goal fact sheet is intended as an aid to assist in the lifestyle counselling of hypertensive patients. Patient Lifestyle changes and the effect on Blood pressure are listed on the first side. The other side has Blood Pressure Basics: one page patient education piece on blood pressure that outlines definitions and management. No major changes to the previously ethically approved version except updating information based on the new CHEP guidelines.	Pharmacists

**Patient Toolkit**	1."Take the Pressure Off' Resource Book	Evidence-informed patient HBP education workbook will be provided to all patients. It will serve as a knowledge resource utilizing easy language. This resource will also serve as an identification tool for the pharmacist to initiate lifestyle counselling with the enrolled and consented patients. It is consistent with the latest CHEP recommendations. There are 3 key components of this patient resource book: i) An easy to read resource book designed to provide your patients with a basic understanding of high blood pressure and to promote self-management ii) Patient-Provider Agreement: A tool intended to promote conversations and concordance between patients and you, as well as patient's decision-making. iii) iii) Patient Log Book: An interactive tool that promotes self-management among patients. It enables monitoring of their progress for 3 key co-morbidities: hypertension; diabetes; dyslipidemia, and tracking of medications and questions/topics they wish to follow-up with different members of the healthcare team.	Patient

	4. Blood Pressure Action Plan	HSFO online or phone-in service that asks an individual questions regarding lifestyle and risk factors and offers a personalized plan with realistic strategies for living a healthier life. Individuals may also opt into an email support program that sends periodic email messages to help the individual through the stages of change. Previously ethically approved and no changes has been introduced.	General public patients who enter the Initiative will be referred to BPAP.

The patient will also be offered the patient toolkit, including the "Take the Pressure Off" education/resource book. This patient resource provides information and education on hypertension, its risk factors, lifestyle change information, and lists other resources. The Patient-Provider Agreement is incorporated into the "Take the Pressure Off" book and is to be reviewed and completed during the initial enrolment visit, including the BP goal, the patient's selected lifestyle risk factor which (s)he wishes to focus on in the coming months/years and the patient's personal plan of action for this selected lifestyle risk factor. Documentation of the BP goal and patient selected lifestyle risk factor in the HBP Flow sheet is also done.

**Figure 2:**
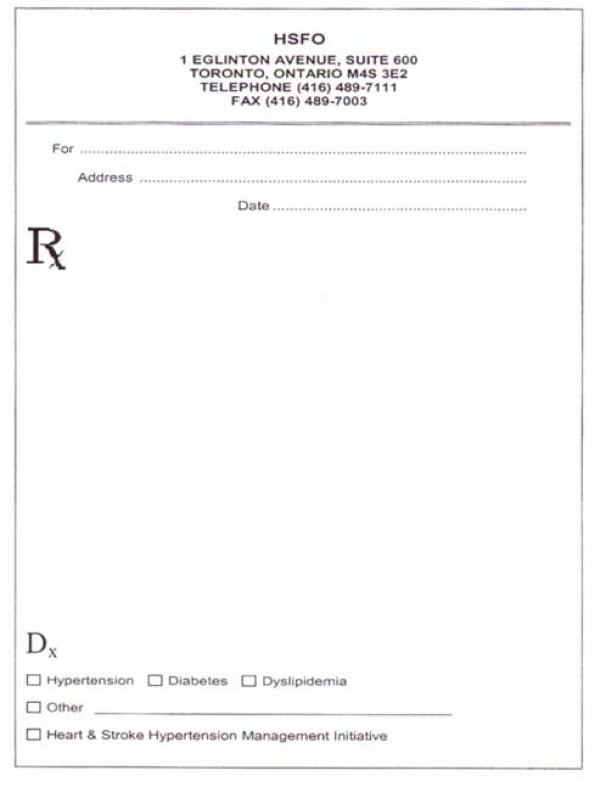
**Rx Dx Prescription**.

Prescriptions are written using an 'Rx Dx' prescription (seen in figure 2), in which any diagnoses of hypertension, dyslipidemia or diabetes can be identified at the bottom of the prescription. This enables the communication of these diagnoses to the patient's pharmacist, who can then support and reinforce the key messages and offer further lifestyle and medications counselling to the patient. A patient counselling tool, the "Reach Your Goal of BP Control" fact sheet, is provided to the pharmacist, for use in patient counselling.

An office automated blood pressure monitor, the BpTRU, is provided to each family physician and nurse practitioner. The BpTRU monitor is to be used to take the blood pressure reading [6]. As data from each healthcare provider (physicians and nurse practitioners) is inputted in the web based data set, confidential practice audit reports will be produced for each healthcare provider. The HMI's supporting practice and behavior change tools that are to be integrated into practice will be presented during the Launch Workshop.

##### Assignment to Immediate Intervention and Delayed intervention groups

Assignment to the immediate intervention and delayed intervention groups will be conducted at the site level rather than at the practitioner level or patient level. This is to mitigate the potential risk at a site of "contamination" of the comparison by practitioners sharing tools and information about hypertension management (as well as patients), if some practitioners at a site were assigned to the immediate intervention group and some to the delayed intervention group.

Initial consideration was given to randomizing the groups, however with only 11 sites, randomization posed risks of introducing bias. Sites in neighbouring communities could have cross contamination; the diverse and vast geography of the Province of Ontario must be accounted for, particularly because of the more rural north and the more densely populated south. Also, approximately half of the clinics had adopted an electronic medical record at study start, indicating the potential for the more rapid adoption of new techniques. With only 11 sites and too many variables to stratify by to allow randomization, in order to ensure that the sites in the immediate intervention and delayed intervention groups are not different from each other, the sites will be assigned to the intervention groups manually to ensure a distribution of the variables as evenly as possible. Allocation of sites into immediate intervention and delayed intervention groups will also take into consideration geographic proximity between sites. Sites in neighbouring communities will be allocated to the same grouping since communication between participants in proximal groupings could potentially contaminate comparisons.

Demographic and baseline data will be compared between the immediate intervention and delayed intervention groups to determine if the groups did differ significantly in terms of healthcare provider practice patterns, site characteristics, and patient blood pressure control before the implementation of the program. If any statistically significant differences are detected between immediate intervention and delayed intervention groups, then these will be taken into account in analyses following the immediate intervention. The immediate intervention group will have the intervention for a longer duration than the delayed intervention group, providing an opportunity to determine whether there is a "dosage effect" associated with having the immediate intervention for a longer time period.

### Statistical Design and Analysis

The standard deviation used to calculate sample size is derived from results of the alpha phase. In order to have power of 0.9 to detect a difference of 5 mmHg with beta = 0.20 and alpha = 0.05, 337 patients would be required in each group.

Descriptive statistics will be calculated for all variables of interest. Continuous measures will be summarized using means and standard deviations whereas categorical measures were summarized using counts and percentages. For univariate analyses, two sample two-sided t tests will be using for continuous data, whereas chi-square analyses (or Fisher's exact tests in the case of cell sizes) will be used for categorical data. Regression analyses will be constructed from covariates known to impact on blood pressure. All analyses will be carried out using SAS Version 9.1 (SAS Institute, Cary, NC, USA).

#### Patient Recruitment Strategy and Consent

Shortly before attending the Launch Workshop, the healthcare providers will identify potential eligible patients for the HMI Beta Phase, based on identified inclusion and exclusion criteria. Each full time equivalent rostering physician and nurse practitioner is requested to recruit a minimum of 100 patients and up to a maximum of 200 patients. A patient friendly consent form has been developed and will be signed by each participant. Copies of the consent, an accompanying script and other materials will be provided to each participating office. In addition to these documents, an implementation guide will also be provided to each site. (Thorough explanations of the materials will also be provided by Heart and Stroke Outreach and Practice Support Specialists at 'on site' visits.)

Appointments with the potential eligible patients will be scheduled to occur subsequent to the workshop with the participating practitioners. During this visit, these potential eligible patients will be provided with information about the HMI Beta Phase, patient consent will be requested and documented and a baseline assessment including physical measures conducted and documented. When a patient provides consent, (s)he is deemed to have enrolled in the HMI Beta Phase. Ethics approval has been obtained from the Sunnybrook Health Sciences Centre Research Ethics Board (approval #: 474-2006).

Utilizing adult learning best practices, the format of the site initiation Launch Workshop is one in which physicians, pharmacists and nurses will learn together and discuss barriers and solutions to better manage hypertension together and via small group breakout sessions, develop a community practice plan to improve the management and control of hypertension among their patients. The purposes of this interactive, inter-professional workshop include addressing any knowledge gaps identified in the healthcare provider needs assessment, introducing concepts of systems change theory and related applications, and developing approaches to high blood pressure based on inter-professional teamwork. This will be accomplished by providing information/education on best practices in management of hypertension, and a tool kit of supporting practice and behaviour change tools to all participating healthcare providers.

#### Implementation of Toolkit and Interventions

When a patient provides consent, (s)he is deemed to have enrolled in the HMI Beta Phase. Only then will his/her baseline data collected on the registration (baseline) visit data form designed as a flow sheet, be entered into the web-based dataset, using a unique ID to ensure that the web-based dataset is anonymized and pseudonymized. A flow sheet form will continue to be used during the subsequent patient visits. The flow sheets will become a part of the patient's chart. At the time of use, the flow sheet will graphically show the trends from all earlier visits in several key variables including systolic/diastolic blood pressure, BMI/waist circumference, lipids and lifestyle goal variables.

At the time of recruitment, the sites in this initiative were divided almost 50-50 in the use of either paper or electronic medical record (EMR) for clinical/patient record documentation. Existing EMR systems at the sites are unable to systematically and fully capture and subsequently extract and upload the patient data required for this project. Therefore, each participating EMR vendor has undertaken to create and insert into their system a "data collection template screen" specifically customized to capture and export the data set requirements of this project. After a patient has consented, a copy of the patient information needed for this strategy is exported from the practitioner's EMR and transferred securely into the project's central, securely housed clinical data repository ("web-based dataset"). Specifically, the data will be formatted in a standardized project-specific format as an XML file, along with enforcement of XSD-based validity checks, and transmitted to the web-based data set via HTTPS (encrypted HTTP) using a SOAP-based web service provided by the database host. The aforementioned encryption will prevent access to patient information while it is in transit. For electronic medical record (EMR) sites, the data will automatically be exported once nightly in a standardized format from the site's EMR to the web-based dataset.

For paper-based sites, the initial paper flow sheet is provided to each healthcare provider with a paper based clinical record documentation system. Follow up visit paper flow sheets are obtained by downloading the follow-up visit form from the website shortly before every follow-up visit, i.e. after the prior visit's data have been processed and incorporated into the updated data graphs. Two methods are available for transferring the data from the paper forms to the web-based data set. In the first method, at the end of each visit, the flow sheet will be faxed by toll-free number to the data centre for optical scanning and aggregation into the web-based data set. To insure data accuracy, the scanned information will be validated by a human operator at the centre and corrected for errors. The faxed form will include neither the name of the patient nor any other identifying information such as address or phone number. This is possible because each patient will be assigned a unique sequential identifier by the practitioner's office at the time of the baseline (registration) visit, the identifier being pre-printed on the practitioner's pad of blank Registration visit forms. The practitioner's office will subsequently supply the patient's name directly into the web-based dataset by matching with the anonymous identifier and birthdate. This procedure facilitates the subsequent downloading of each patient's own follow-up forms containing their pre-printed data graphs, whose ongoing feedback is an important component to promoting the success of the project. In the second method, at the end of each visit, the data from the flow sheet will be manually entered into a secure website directly linked to the web-based data set.

After patient information is entered into the dataset, monthly and quarterly confidential practice audit reports will be available to all practitioners on the secure website linked to the web-based dataset. Moreover, the practitioner will be able to access the data set at anytime and generate ad hoc "drill-down" reports on his/her patients only. While the same practitioner can compare aggregate data from different sites, he/she cannot access the individual patient information of another physician. The web-based dataset will generate regular confidential practice reports based on the data submitted, for each participating physician and nurse practitioner, comparing the practice to that of other providers at the same site and to those of the other sites and participating healthcare providers. Reports are also generated at a site level for the other healthcare providers to access.

The web-based dataset will be hosted on a Personal Health Information Protection Act (PHIPA) compliant server provided by the dataset developer.

The patient will also be offered the patient toolkit, including the "Take the Pressure Off" patient book. This patient resource provides information and education on hypertension, its risk factors, lifestyle change information, and lists other resources. The Patient-Provider Agreement will be reviewed and completed, including the BP goal, the patient's selected lifestyle risk factor which (s)he wishes to focus on in the coming months/years and the patient's personal plan of action for this selected lifestyle risk factor. The Patient-Provider Agreement is incorporated into the "Take The Pressure Off". The BP goal and patient selected lifestyle risk factor is also documented in the HBP Registration (baseline) and Flow sheet (follow-up) data forms.

In order to assess the impact of the Hypertension Management Initiative on both healthcare provider practice patterns and patient blood pressure readings and control, it is necessary to compare data from the pre-program period to the program period. In order to assess any changes from before to after the intervention, a combination of chart reviews, for the pre-program period, and the web-based patient dataset, for the program period, will be used. Thus, data about the practice patterns of individual healthcare providers and their individual patients' blood pressure data will be used as their own comparators pre-intervention versus post-intervention.

Given that HSFO approached this particular group of health care providers to participate in a program relating to hypertension, this may have heightened their awareness of the issue and affected their management of patients with hypertension (in addition to the influence of the intervention itself). This phenomenon in which subjects of research may be affected by the knowledge that they are subjects of research is known as the Hawthorne Effect. For this reason, data will be collected from both the immediate intervention and delayed intervention groups pertaining to office visits conducted during the six months prior to initial contact with sites for recruitment into the initiative as well as data from office visits after first contact but before the site initiation workshop. Gathering this data will allow an assessment of practice patterns for these groups between the pre-recruitment period and the period following recruitment, which may relate to the Hawthorne Effect. As well, this will allow comparisons to be made between the immediate intervention and delayed intervention groups in terms of patient blood pressure readings as well as healthcare provider practice patterns.

In the patient consent form, patients are informed that information from their medical chart may be collected in order to evaluate the education and tools provided to themselves and their health care provider, and that all information collected would be kept strictly confidential and would be grouped for analysis. All participating and nurse practitioners have provided written consent to a chart review with the intent to evaluate the education and tools provided to the healthcare providers.

## Competing interests

The authors declare that they have no competing interests.

## Authors' contributions

All authors helped to conceive of the study, and participated in its design and coordination and helped to draft the manuscript. All authors read and approved the final manuscript.

## Pre-publication history

The pre-publication history for this paper can be accessed here:



## References

[B1] Health Canada (2002). Economic Burden of Illness in Canada, 1998.

[B2] Joffres MR, Hamet P, Rabkin SW, Gelskey D, Hogan K, Fodor G (1992). Prevalence, control and awareness of high blood pressure among Canadian adults. Canadian Heart Health Surveys Research Group. CMAJ.

[B3] Joffres MR, Hamet P, MacLean DR, L'italien GJ, Fodor G (2001). Distribution of blood pressure and hypertension in Canada and the United States. Am J Hypertens.

[B4] Khan NA, Hemmelgarn B, Padwal R, Larochelle P, Mahon JL, Lewanczuk RZ (2007). The 2007 Canadian Hypertension Education Program recommendations for the management of hypertension: part 2 – therapy. Can J Cardiol.

[B5] Khan NA, Hemmelgarn B, Padwal R, Larochelle P, Mahon JL, Lewanczuk RZ (2007). The 2007 Canadian Hypertension Education Program recommendations for the management of hypertension: part 2 – therapy. Can J Cardiol.

[B6] Mattu GS, Heran BS, Wright JM (2004). Overall accuracy of the BpTRU – an automated electronic blood pressure device. Blood Press Monit.

